# 

**Published:** 1817-10

**Authors:** 


### CORRESPONDENCE.

Dr. Kennedy's Description of an Ossiform Substance developed in the Vulvo-labial Texture, has been received, and will appear in our next number.

Dr. Palmer's Case of Gangrene of the Lungs will be published in the December number.

The Editors have to regret the necessity of postponing, for the present, the interesting Case of Fractured Cranium, by Dr. Dobson: that gentleman's favours will always be received with peculiar pleasure.

Reviews of Marshall on Arsenic, and Mayow on Insanity, are ready, and waiting insertion.

☞ Authors and Publishers are requested to send Copies of new Works for Review as early as possible after their publication. They will be reviewed early in proportion.

